# Genetic Code Expansion System for Tight Control of Gene Expression in *Bombyx mori* Cell Lines

**DOI:** 10.3390/insects12121081

**Published:** 2021-12-01

**Authors:** Wei Lu, Ruolin Wang, Pan Wang, Sanyuan Ma, Qingyou Xia

**Affiliations:** 1 State Key Laboratory of Silkworm Genome Biology, Biological Science Research Center, Southwest University, Chongqing 400715, China; luw10@outlook.com (W.L.); wangrl_1203@126.com (R.W.); wp060510@163.com (P.W.); 2 Chongqing Key Laboratory of Sericulture Science, Chongqing Engineering and Technology Research Center for Novel Silk Materials, Chongqing 400715, China

**Keywords:** *Bombyx mori*, gene expression, genetic code expansion system

## Abstract

**Simple Summary:**

*Bombyx mori* is a lepidopteran insect with economic value. Its genetic background is clear, and genome sequence is relatively complete, but the function of many genes has not been determined. The genetic code expansion system has become an important means of gene function research. In this study, a genetic code expansion system suitable for *B. mori* cells was established. This system included a modified tRNA^Pyl^/Pyrrolysyl-tRNA synthetase (PylRS) pair from *Methanosarcina mazei*, the reporter gene D[TAG]G formed by DsRed and EGFP through amber stop codon TAG connection and the unnatural amino acid H-Lys(Boc)-OH. In silkworm BmE and BmNs cell lines, the reporter gene expression was strictly controlled by H-Lys(Boc)-OH in the presence of both PylRS and tRNA^Pyl^. The silkworm genetic code expansion system established here is another useful controllable gene expression system besides tetracycline induced expression system.

**Abstract:**

Inducible gene expression systems are important tools for studying gene function and to control protein synthesis. With the completion of the detailed map of the silkworm (*Bombyx mori*) genome, the study of *Bombyx mori* has entered the post-genome era. While the functions of many genes have been described in detail, many coding genes remain unidentified. Except for the available tetracycline induction system, there is currently a dearth of other effective induction systems for *B. mori*. A genetic code expansion system can be used for protein labeling and to regulate gene expression. Here, we have established a genetic code expansion system for *B. mori* based on the well-researched tRNA^Pyl^/PylRS pair from *Methanosarcina mazei*. We used H-Lys(Boc)-OH, which is a lysine derivative to efficiently and tightly control the expression of the reporter gene DsRed[TAG]EGFP (D[TAG]G), which encoded a H-Lys(Boc)-OH-bearing protein fused with DsRed and EGFP (here regarded as D[Boc]G) in *B. mori* cell lines BmE and BmNs. In D[TAG]G, the amber stop codon is recognized as the orthogonal tRNA^Pyl^. Successful application of genetic code expansion system in silkworm cell lines will support the research into the function of silkworm genes and paves the way for the identification of new genes and protein markers in silkworm.

## 1. Introduction

Gene expression is one of the essential mechanisms underlying life and is regulated by the intracellular and extracellular environment. Based on the spatiotemporal differences in expression, the genes can be divided into constitutive and inducible genes. Constitutive genes are not pronouncedly impacted by changes in the environment, and their products are often indispensable for the whole life process, while inducible genes are extremely susceptible to environmental change and are highly expressed upon stimulation by specific signals.

Gene expression can be induced in various ways, including by chemical substances [1,2,3,4,5], temperature [6,7], light [8,9,10,11], and others. These methods regulate gene expression at the transcriptional level. In addition, gene expression can be effectively controlled by controlling protein synthesis and degradation [3,12,13].

In nature, nearly all organisms use the same genetic code. Generally, except for the start codon (ATG) and the stop codons (TAA, TAG, and TGA), the remaining 61 codons encode natural amino acids [14]. However, some bacteria [15,16], mycoplasma [17], and yeast mitochondria [18,19] use the UGA codon to encode tryptophan. Further, some bacteria use tRNA/aminoacyl-tRNA synthetase (aaRS) to recognize stop codons, as well as introduce unnatural amino acids (UAAs) into proteins [20,21,22]. Accordingly, based on these observations, the tRNA/aaRS pair has been developed to establish orthogonal genetic code expansion systems suitable for different organisms [23,24,25,26,27,28,29]. tRNA^Pyl^/PylS from *Methanosarcina mazei* is the most widely used because it shows orthogonality in both prokaryotes and eukaryotes and can be developed to specifically recognize different UAAs [30]. Therein, PylRS catalyzes the formation of aminoacyl tRNA from UAAs and tRNA, which is then recognized by the ribosome, with UAAs incorporated into protein. This system can introduce many UAAs with different functional groups into the protein of interest for fluorescent labeling of proteins, control of protein activity [31,32,33,34,35,36]. In addition to these applications, Ma et al. [13] established an orthogonal expanded Genetic code system in the human HEK293 cell line to control clustered regularly interspaced short palindromic repeats/Cas9, thereby reducing the off-target efficiency of Cas9.

*Bombyx mori, B. mori* is an economically important insect with a breeding history of more than 5000 years in China. It is a lepidopteran insect whose genome has been extensively studied, with the insights gained applied to pest control [37]. However, because of the long rearing cycle, studying gene function on *B. mori*-individual level is cost-intensive. Insect cell line is an alternative for studying gene function and signaling pathways because of the short culture cycle and low cost. The commonly used *B. mori* cell lines are an embryonic cell line, BmE, and ovary cell lines, BmNs, and BmN4. To date, few studies on inducible gene expression in *B. mori* cells have been published. A popular system involves using tetracycline to control the turning-on and -off of target genes and is mainly used to regulate gene transcription. To the best of our knowledge, an effective tool for controlling gene expression in *B. mori* cells at the protein level is lacking.

In the current study, we constructed pylT and pylS transient expression vector and a reporter vector harboring the reporter gene D[TAG]G, suitable for BmE and BmNs cell lines. In the reporter vector, the red fluorescent protein (*DsRed*) gene, ending with an amber stop codon (TAG), from *Discosoma striata* is followed by enhanced green fluorescent protein (*EGFP*) gene. After transiently transfecting cells with the constructs, the expression of DsRed and EGFP fusion protein is controlled by the addition of UAA. The successful application of the genetic code expansion system in *B. mori* cells lays the foundation for studying the function of *B. mori* genes, especially toxic genes, and provides an effective means for identifying new coding genes, accurately regulating spatiotemporal gene expression, and labeling economically valuable silk fibroin proteins.

## 2. Materials and Methods

### 2.1. Plasmids

The genetic code expansion system established herein includes the *pylT* and *pylS* genes from *M. mazei*, and the reporter gene D[TAG]G. To implement it, silkworm *U6* promoter and *pylT* gene were synthesized into pUC57 plasmid to constitute the vector pUC57-U6-pylT (herein abbreviated as pylT) by Genscript (Nanjing, China). Based on the codon usage frequency of *B. mori*, the *pylS* gene sequence was optimized, and synthesized into pUC57 plasmid by Genscript (Nanjing, China). Then, pUC57-pylS and pMD19-hA4-dCas9-Ser1pA plasmids (a gift from Dr. Yuanyuan Liu from State Key Laboratory of Silkworm Genome Biology) were digested using *Spe* I and *Hin*d III. They were then ligated to form pMD19-hA4-pylS-Ser1pA (herein abbreviated as pylS). The reporter gene D[TAG]G is a fusion of *D. striata DsRed* and *EGFP* genes. Firstly, pEasy-T1-DsRed from T-cloning was digested by *Spe* I and *Hind* III and connected with pMD19-hA4-dCas9-Ser1pA linearized by *Spe* I and *Hind* III to form an intermediate vector pMD19-hA4-DsRed-Ser1pA. Then, the pMD19-hA4-DsRed-Ser1pA and pEasy-T1-EGFP vectors from T-cloning were linearized with *Sph* I and *Hind* III and ligated to form the final vector pMD19-hA4-D[TAG]G-Ser1pA (Table 1 and Table S1).

### 2.2. Cell Transfection

*Bombyx mori* cell lines BmE and BmNs used in the current study were from the State Key Laboratory of Silkworm Genome Biology. The cells were grown on Grace’s insect medium (Gibco, NY, USA, 11595030) and TC-100 insect medium (Sigma, St. Louis, MO, USA, T3160) supplemented with 10% (*v*/*v*) fetal bovine serum at 27 °C. Transfection of BmE cells was performed when the density of adherent *B. mori* cells was approximately 80%. For the transfection, 50 μL of serum-free Grace’s insect medium was mixed with 3 μL of Lipofectamine 2000 transfection reagent (Invitrogen, Carlsbad, CA, USA, YZ-11668) and a total of 2.0 μg plasmids (pUC57-U6-tRNA, pMD19-hA4-pylS-Ser1pA, and pMD19-hA4-D[TAG]G-Ser1pA). The mixture was then incubated at 25 °C for 20 min. The cells were washed twice with serum-free Grace’s insect medium, and the mixture of the transfection reagent and plasmids was added to each well. The cells were incubated at 27 °C; 6–8 h after the transfection, the medium was replaced with Grace’s insect medium containing 10 mM H-Lys(Boc)-OH (Chemical Abstracts Service [CAS] registry number 2418-95-3, BACHEM, 1054762). BmNs cells were transfected in a similar manner, except that the TC-100 insect medium was used.

### 2.3. Cell Imaging

Three days after transfection, the cells were washed with 1X Phosphate Buffered Saline with Tween® 20 (PBST, 8 mM Na_2_HPO_4_, 0.136 M NaCl, 2 mM KH_2_PO_4_, 2.6 mM KCl, 0.05% (*v*/*v*) Tween-20), three times, and fixed in 4 % Paraformaldehyde Fix Solution (Beyotime, Shanghai, China, P0099-100 mL) at 25 °C for 10–15 min. The cells were washed three times with 300 μL 1X PBST. Then, 100 μL of 4′,6-diamidino-2-phenylindole (DAPI, Invitrogen, Carlsbad, CA, USA, D1306) working solution were added, and the samples were incubated for 10 min at 25 °C in the dark. The cells were washed again with PBST, three times every 5 min. Finally, the cells were imaged using fluorescence microscope (Olympus, Tokyo, Japan) at different magnifications. The used excitation wavelengths for DAPI, DsRed, and EGFP were 405 nm, 488 nm, and 559 nm, respectively. The cells were also imaged using the confocal microscope (Olympus, FV1000, Tokyo, Japan). 

### 2.4. Western Blotting

Protein was extracted from cells 3 d after transfection, as follows. The cells were washed twice with phosphate-buffered saline pre-chilled to 4 °C. Then, 100 μL NP-40 lysis buffer (Beyotime, Shanghai, China, P0013F) and 1 mM phenylmethylsulfonyl fluoride (PMSF, YEASEN, Shanghai, China, 20104ES03) were added to wash off the adherent cells. The samples were centrifuged at 13,000× *g* for 5 min at 4 °C, and the supernatant was transferred to a new 1.5-mL centrifuge tube pre-chilled at 4 °C. The collected cell lysate was immediately assayed by Western blotting or stored at −20 °C.

After determining cell lysate concentration using a BCA Protein Assay Kit (Beyotime, Shanghai, China, P0012), 50 μg protein was separated by 10% sodium dodecyl sulfate-polyacrylamide gel electrophoresis (SDS-PAGE) and transferred to polyvinylidene fluoride membrane (PVDF, GE Healthcare, Freiburg, Germany). The following primary antibodies were used: anti-DsRed antibody (Biovision, Tucson, AZ, USA, 3993-100) and anti-GFP (Biovision, 3999-100). The following secondary antibodies were used: Goat anti-Rabbit IgG (H + L) Secondary Antibody (Invitrogen, Carlsbad, CA, USA, 31460), and Goat anti-Mouse IgG (H + L) Secondary Antibody (Invitrogen, 31430). The protein signal was detected by Pierce enhanced chemiluminescence (ECL) reagents (Thermo Fisher Scientific, Waltham, MA, USA, 32106).

## 3. Results

### 3.1. Strategy for a Novel Genetic Code Expansion System to Control Gene Expression in B. mori Cell Lines

**Figure 1:**
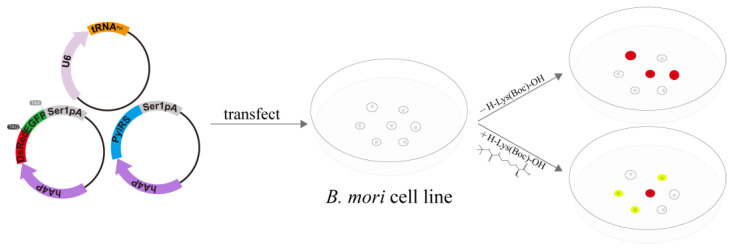
Schematic diagram of UAA-induced gene expression in *B. mori* cells. The approach includes construction of genetic code expansion system elements, transient transfection of *B. mori* cells, and cell imaging. Cell imaging and Western blotting are performed 3 d after transfection.

Since its establishment, the tRNA^Pyl^/PylRS-based genetic code expansion system has been constantly developed and evolved [38]. To determine whether this genetic code expansion system can be used to control gene expression in *B. mori* cells, we selected two *B. mori* cell lines. We constructed a vector where the *pylT* expression is controlled by *U6* promoter; a pylS expression vector, with the *pylS* gene sequence optimized based on the codon usage frequency in *B. mori*; and a reporter vector harboring the D[TAG]G gene (*DsRed* gene harboring the amber stop codon ligated at the 5′ end of the *EGFP* gene) under the control of the broad-spectrum promoter *A4* (Figure 1). We then co-transfected the BmE and BmNs cells with the three vectors, and added H-Lys(Boc)-OH to the incubation medium 8 h after the transfection to control the expression of the reporter gene. Three days after the transfection, we analyzed the expression of the reporter gene using confocal microscopy and Western blotting, as described below.

### 3.2. Testing of the Novel Genetic Code Expansion System in BmE Cell Line

**Figure 2:**
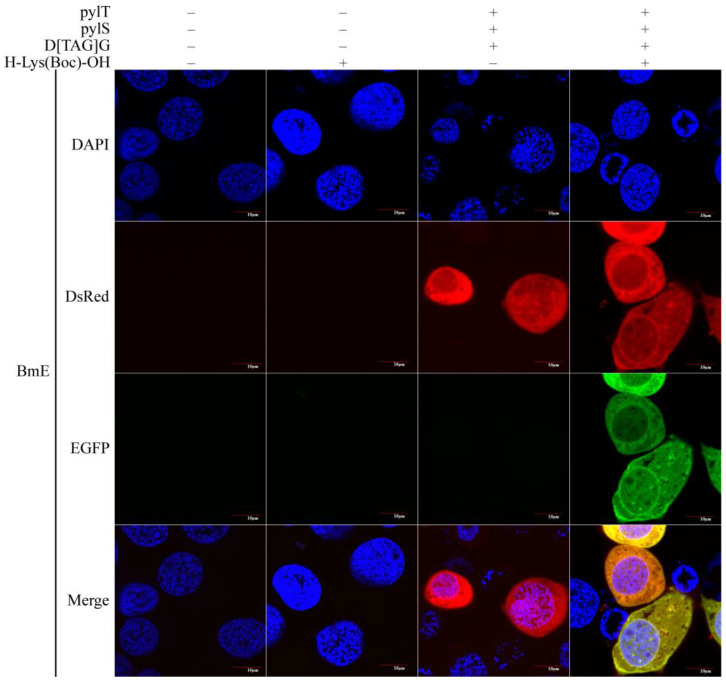
Expression of the reporter gene in BmE cells under the control of H-Lys(Boc)-OH. In the system, the reporter gene D[TAG]G is only expressed in the presence of pylT and pylS. For the imaging, cell nucleus was stained with DAPI. When the excitation wavelengths of 488 nm and 559 nm were used, both red and green fluorescence was observed in BmE cells. In the absence of H-Lys(Boc)-OH, only red fluorescence was observed. “−” and “+” indicate the absence and presence (of plasmid or H-Lys(Boc)-OH), respectively.

Many *B. mori* cell lines are available. Among them, BmE cells are widely used because of their rapid growth and good adhesion properties. Therefore, we tested the *B. mori* genetic code expansion system with orthogonal tRNA^Pyl^/PylRS pair in BmE cells. The cells were transfected and imaged as described in Methods. Cells with green fluorescence were not detected in the absence of H-Lys(Boc)-OH and were detected only when H-Lys(Boc)-OH, D[TAG]G, pylT, and pylS were present. In the cells, red and green fluorescence signals were dispersed throughout the cytoplasm (Figure 2). A BmE cell is short, spindly, or round, and the nucleus is round or nearly round. In fluorescent cells, the nucleus was clearly visible, as the reporter gene is transcribed in the nucleus and the mRNA translated in the cytoplasm. These observations indicated that the genetic code expansion system established in the BmE cell line could be used to control exogenous gene expression in these cells by the provision of UAA.

### 3.3. Application of the Novel Genetic Code Expansion System in BmNs Cell Line

**Figure 3:**
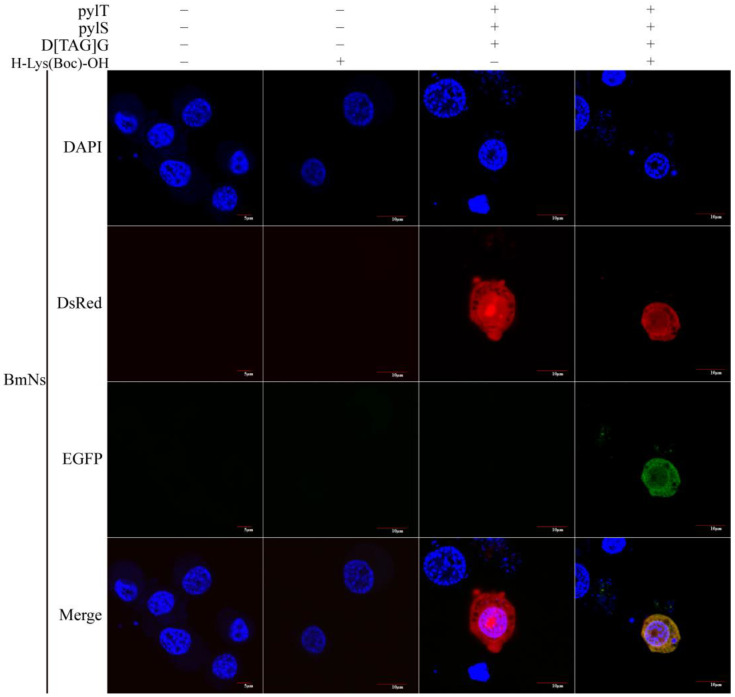
Expression of the reporter gene in BmNs cells under the control of H-Lys(Boc)-OH. In the system, the reporter gene D[TAG]G is only expressed in the presence of pylT and pylS. For the imaging, cell nucleus was stained with DAPI. When the excitation wavelengths of 488 nm and 559 nm were used, both red and green fluorescence was observed in BmNs cells. In the absence of H-Lys(Boc)-OH, only red fluorescence was observed. “−” and “+” indicate the absence and presence plasmid or H-Lys(Boc)-OH), respectively.

The *B. mori* ovary cell line BmNs grows rapidly, and the cells are round, short, spindly, or polygonal. To determine the versatility of the *B. mori* genetic code expansion system established in the BmE cell line, we co-transfected BmNs cells with the pylT, pylS, and D[TAG]G constructs, as for the BmE cells. As determined by confocal microscopy 3 d after the transfection, in the absence of H-Lys(Boc)-OH, the cells did not fluoresce or only some cells with red fluorescence were detected. In the presence of H-Lys(Boc)-OH, D[TAG]G, pylT, pylS, BmNs cells exhibited red and green fluorescence (Figure 3). In these cells, the fluorescence was distributed throughout the cytoplasm, with the nucleus visible, which was consistent with the observations for BmE cells. These findings suggested that the established genetic code expansion system was not only applicable to BmE cells, but also to other cell lines.

### 3.4. Efficient and Orthogonal Bombyx mori Genetic Code Extension System

**Figure 4:**
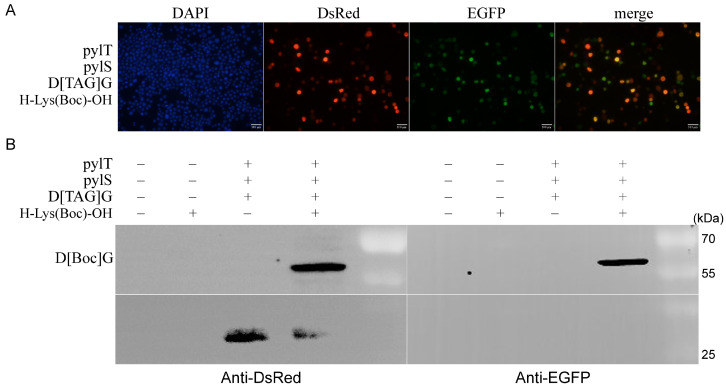
Efficiency and orthogonality of the genetic code expansion system. (**A**) BmNs cell imaging at low magnification. In the presence of pylT, pylS and H-Lys(Boc)-OH, the reporter gene D[TAG]G was successfully expressed, and some BmNs cells with red and green fluorescence were discovered. (**B**) Western blotting assay of fusion protein D[Boc]G. The BmNs cells were collected, and protein was extracted 3 d after transfection. The D[Boc]G protein was detected using anti-DsRed and anti-EGFP antibodies. The fusion protein D[Boc]G was only detected in the presence of pylT, pylS and H-Lys(Boc)-OH. Only DsRed was detected in the absence of H-Lys(Boc)-OH. “−” and “+” indicate the absence and presence plasmid or H-Lys(Boc)-OH), respectively.

For the excellent orthogonality and introduction efficiency, the genetic code expansion system has been widely used. Here, the orthogonality and introduction efficiency of the silkworm genetic code expansion system were evaluated by fluorescence imaging combined with Western blotting. According to the procedures in the methods, we extracted the BmE and BmNs cells 3 d after transfection and selected the areas with dense cells for fluorescence imaging. Both H-Lys(Boc)-OH and genetic code amplification system components were present, the cells with red and green fluorescence could be found, and the cells with green fluorescence almost overlapped with red fluorescent cells (Figure 4A and Figure S1). This implied that H-Lys(Boc)-OH was introduced into D[Boc]G fusion protein in almost every fluorescent cell.

In silkworm, the amber stop codon in the reporter gene D[TAG]G was recognized by the exogenous tRNA^Pyl^ and also by the endogenous eRF1, to terminate the translation of the polypeptide. Therefore, we speculated that those cells with red fluorescence were derived from the fusion protein D[Boc]G and also from the DsRed protein. To test this, 3 d after the transfection, we analyzed BmNs cells by Western blotting. We first probed the extracted protein with an anti-DsRed antibody. Regardless of the presence of H-Lys(Boc)-OH, in cells harboring the reporter gene, an approximately 27-kDa protein band was detected, which corresponded to the size of DsRed. In the presence of H-Lys(Boc)-OH and the genetic code expansion system elements, an approximately 53-kDa band was detected, which was about 74% of the 27-kDa protein band. Indeed, probing with the anti-EGFP antibody confirmed that the 53-kDa protein band was the fusion protein D[Boc]G. These results suggested that the genetic code expansion system established here was highly efficient and orthogonal in silkworm cells (Figure 4B and Figure S2).

## 4. Discussion

With the completion of the *B. mori* genome project, silkworm research has entered the post-genome era. To take advantage of these developments, ideally, the expression of endogenous and exogenous genes in *B. mori* could be controlled temporally and spatially using a suitable inducible expression system, paving the way for understanding the role of genes in insect growth and metamorphosis. In the current study, we established a novel genetic code expansion system suitable for *B. mori*, to control the expression of the reporter gene D[TAG]G in BmE and BmNs cells in response to UAA availability.

In addition to our genetic code expansion system, the tetracycline induced expression (Tet-On) system of silkworm has been established previously, which is mainly composed of reverse tetracycline transcriptional activator (rtTA) and Tet-responsive element (TRE) containing multiple repeated TetO sequences [39]. Compared with the Tet-On system, the Genetic code extension system presented herein has two major advantages. Firstly, it is versatile. Without changing the core elements *pylT* and *pylS*, as long as an amber codon is introduced to the target gene, the gene can be tightly controlled, which is applicable to exogenous genes and endogenous genes, especially spatiotemporal expression genes. However, the Tet-On system needs a mini promoter without an enhancer, not all promoters are available, and it is not friendly to the spatiotemporal expression of endogenous genes. Secondly, it is multi-purpose. The genetic code expansion system can not only control gene expression, but also label and control protein activity. In this study, the tRNA^Pyl^/PylRS pair from *M. barkeri* was used. Due to its good orthogonality and versatility, it is the most popular genetic code extension system [40]. To date, more than 100 structurally and functionally diverse UAAs have been introduced into proteins. The UAAs with fluorophore, azide, or photocaged groups can be used to label, modify protein, or control the activity of the protein. Therefore, in addition to the H-Lys(Boc)-OH, other UAAs should also be effective in silkworm cells.

The incorporation efficiency of UAA is mainly affected by two factors. This first factor is the reporter gene promoter. In the current study, we used the *A4* promoter, which is highly active in *B. mori* cells, to control the expression of *pylS* and the reporter gene D[TAG]G. The second is the endogenous eRF1. As demonstrated by Western blot analysis, the amber stop codon in D[TAG]G is not only recognized by tRNA^Pyl^, which contributes to the formation of the complete fusion protein D[Boc]G, but also by *B. mori* eRF1, which affects the yield of D[Boc]G. To address this issue, Johnson et al. [41] knocked out the bacterial RF1 gene to improve the efficiency of UAA incorporation into proteins in *E. coli*. Prokaryotes encode two release factors, RF1 and RF2. The former recognizes TAA and TAG, and the latter recognizes TAA and TGA. Since the three stop codons in *B. mori* are all recognized by eRF1, knocking out eRF1 would seriously affect the survival of *B. mori* cells and is not feasible. However, inspired by the experiments of Johnson et al., replacing the eRF1 of *B. mori* with the prokaryotic RF2 might improve the efficiency of UAA incorporation into proteins.

In fact, in addition to the exogenous gene D[TAG]G, many endogenous genes in almost all organisms harbor the amber stop codon, which allows the incorporation of UAAs into endogenous proteins. Accordingly, Elliott et al. [36] used modified UAAs to label the bacterial proteome, an approach that conferred new functions onto proteins and was also used to identify novel coding genes. Of note, the widespread introduction of UAAs may affect the function of some proteins. Lajoie et al. [42] replaced all the amber stop codons in the bacterial genome with the ochre stop codon and knocked out RF1, which improved the incorporation efficiency of UAAs and did not affect the bacterial growth. The *B. mori* genome, with more than 16,069 protein-coding genes, is approximately 468.3 Mbp [43]. It would be undoubtedly difficult to avoid the impact of UAAs on *B. mori* and other organisms by replacing all amber stop codons with homologous recombination. However, the widely used base editor BE3 can be used to convert cytosine to uracil (C–U) in the target sequence, so as to change the stop codon TAG to TAA [44,45]. This creates the possibility of introducing the amber stop codon into the *B. mori* genome.

In the current study, we used UAA to control the expression of a reporter gene in *B. mori* cell lines. In fact, UAAs can also be absorbed by *B. mori* larva and incorporated into silk fibroin. As early as 2014 and 2016, Teramoto and colleagues [46,47] successfully introduced phenylalanine (Phe) analogs (ClPhe, BrPhe, and AzPhe) at the Phe site of the silk fibroin protein using the modified BmPheRS-α protein, and prepared clickable silk fibroin material. Since the authors have not modified the tRNA^Phe^ and silk fibroin heavy chain gene, these UAAs compete with Phe for tRNA^Phe^ binding. Without a doubt, these Phe analogs could also be introduced into other silk proteins. Appropriately reducing Phe content in the silkworm diet, while increasing the amount of Phe analog in the diet, could increase the Phe analog content in silk fibroin. In Teramoto’s study, both labeled and unmodified silk fibroin proteins were produced. However, using the genetic code expansion system presented herein, the amber stop codon could be introduced into the silk fibroin heavy chain gene, thus enabling the labeling of all silk fibroin proteins.

## 5. Conclusions

In conclusion, we here established a novel genetic code expansion system for use in *B. mori* cell lines. The system is highly efficient, and easy to use, and we anticipate that it will become an important tool in *B. mori* research.

## Figures and Tables

**Table 1:** Primers used in this study.

Gene Name	Primer Name	Sequence (5′ to 3′)
*DsRed*	DsRed-F (*Xba* I+*Spe* I)	GCTCTAGAGGGACTAGTATGGTGCGCTCCTCCAAG
	DsRed-R (*Sph* I)	ACATGCATGCCTACAGGAACAGGTGGTGG
*EGFP*	EGFP-F (*Sph* I)	ACATGCATGCATGGTGAGCAAGGGCGAG
	EGFP-R (*Hin*d III+*Bsr*G I)	CCCAAGCTTTTACTTGTACAGCTCGTCCATG

## Data Availability

Not applicable.

## Supplementary Materials

The following are available online at https://www.mdpi.com/article/10.3390/insects12121081/s1, Table S1: The sequence of *pylS, pylT*, *DsRed* and *EGFP* gene, Figure S1: BmNs cells under the induction of Pyl. (A) BmNs cells were imaged when the reporter gene was absent and pylT, pylS or H-Lys(Boc)-OH was present. (B) BmNs cell imaging in the presence of the reporter gene D[TAG]G. Figure S2: Western blotting assay of fusion protein D[Boc]G.
