# Supplementary material for: Genetic Code Expansion System for Tight Control of Gene Expression in Bombyx mori Cell Lines
Source: Insects. 2021 Dec 1;12(12):1081. doi: 10.3390/insects12121081 (PMC8709394; doi:10.3390/insects12121081)
Supplement: Supplementary file 1 [file insects-12-01081-s001.zip › insects-1451973-supplementary.pdf]

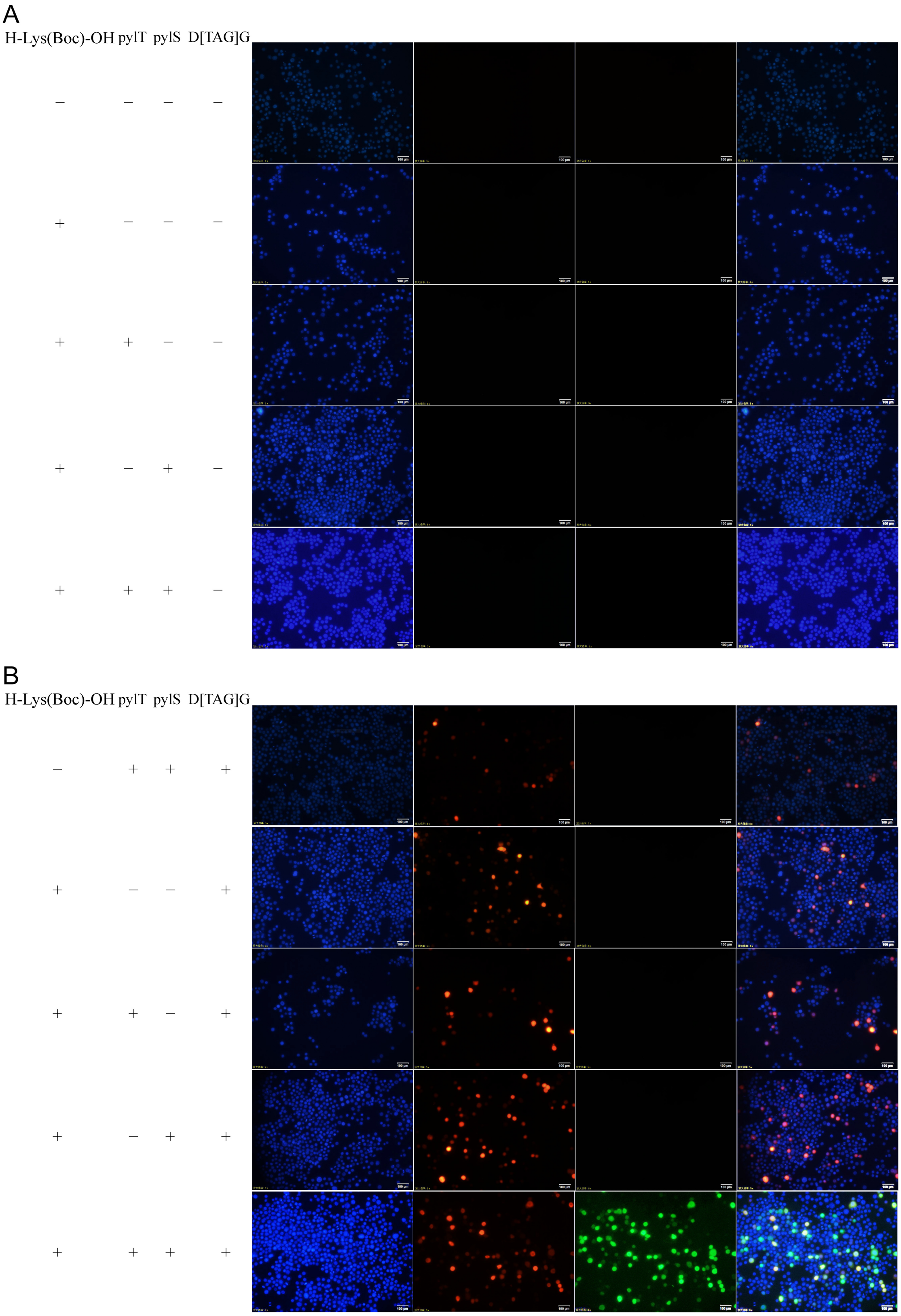

Figure S1. BmNs cells under the induction of H-Lys(Boc)-OH. (**A**) BmNs cells were imaged when the reporter gene was absent and pylT, pylS or H-Lys(Boc)-OH was present. (**B**) BmNs cell imaging in the presence of the reporter gene D[TAG]G.

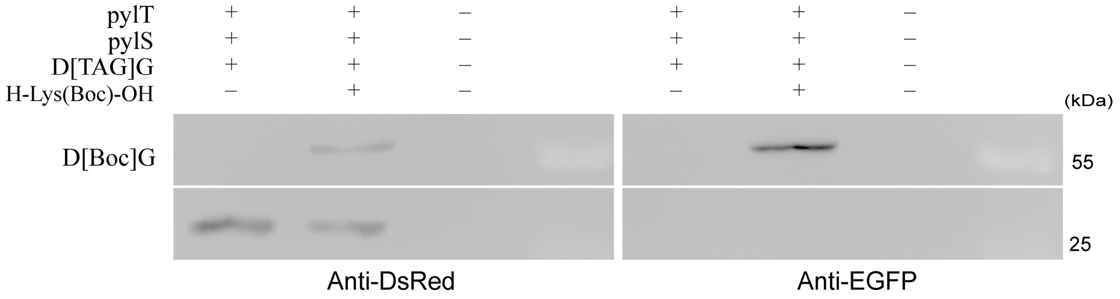

Figure S2. Western blotting assay of fusion protein D[Boc]G. The BmNs cells were collected, and protein was extracted 3 d after transfection. The D[Boc]G protein was detected using anti-DsRed and anti-EGFP antibodies. The fusion protein D[Boc]G was only detected in the presence of D[TAG], pylT, pylS and H-Lys(Boc)-OH. Only DsRed was detected in the absence of H-Lys(Boc)-OH.

Table S1. The sequence of *pylS, pylT, DsRed* and *EGFP* gene.

Note 1 | pMD19-hA4-D[TAG]G sequence

CCGCTCGAGCAGCGTCGTGAAAAGAGGCAATGACAAATACAAAACGACGTATGAG
CAGACCCGTCGCCAAGACGGGTCTACCTCTAAGATGATGTCATTTGTTTTTAAAAC
TAACTCGCTTTACGAGTAGAATTCTACGTGTAAAACATAATCAAGAGATGATGTCA
TTTGTTTTTCAAAACCAAACTCGCTTTACGAGTAGAATTCTACGTGTAAAACACAAT
CAAAAGATGATGTCATTCGTTTTTCAAAACCGAATTTAAGAAATGATGTCATTTGTT
TTTCAAAACCAAACTCGCTTTACGAGCAGAATTCTACGTGTAAAACACAATCAAGA
GATGATGTCATTTGTTTTTCAAAACTGAATGATGTCATTTGTTTTTCAAAACTAAAC
TTGCTTTGCGAGTAGAATTCTACGTGTAAAACACAGTCAAGAGATGATGTCATTTG
TTTTTCAAAACTGAACCGGCTTTACGAGTAGAATTCTACTTGTAAAACATAATCAA
GAGATGATGTCATTTGTTTTTCAAAACTGAACTGGCTTTACGAGTAGAATTCTACGT
GTAAAACATAATCAAGAGATGATGTCATCATTAAACTGATGTCATTTTATACACGA
TTGTTAACATGTTTAATAATGACTAATTTGTTTTTCCAAATTAAACTCGCTTTACGA
GTAGAATTCTACTTGTAACGCACGATTAAGTATGAATCATAAGCTGATGTCATTTGT
TTTCGACATAAAATGTTTATACAATGGAATCTTCTTGTAAATTATCCAAATAATATA
ATTTATCCGATTCTACGTTACATTTAAATTCGTTGTTATCGTACAATTCTTCAGGAC
ACGCCATGTATTGGTCATTTTTAGCGTGCAACCAACGATTGTATTTGACGCCGTCGT
TGGATTGCGTGTTCAGGTTGGCGTACACGTGACTGGGCACGGCTTCTTTTTCCATGG
GACGTCGACTCATCTTGTCACACCTACATCTTACTAATTTCGTAAGTAGATTTTTTTT
TACACGTATAATGTATGTATTCTTTCCTTAATTAACTTATTTTGAAACGAAATAAAT
AGGCTATTAATATTTGGAACTAGGTTGCGGTCAATGTCAATGTCTGTCTCAACTTTA
ATTCAGAATGCCTTGTGTTCCGTAGATGCTATAAATCAATCAAGATGCATCTTGGAT
TGTTGCCAACTCGCAGCTACAAAATTTGTTTCCAAGCCTAAGCATAGTGCTGTACCC
GTTCCCGTGTATTCAAATCCCGTATAATAGTATAATATACTCCGTAAATGTAGTGTC
ACTGCTTGCTGAAATGATATTGCAAGTTCCGTTGGGAATCTTGCCGTTATCAAGCA
ATGCGATATTAGCGGTATGGCGGGAGGGGGACGCGCAGACTCCCTCTGCTGTATTA
CCATATATGGACACAAAACTTCGTGTATTGTACCCTAGCGCGCGATTGGAGGAGAG
TCTGCGGCGGCGGGGCAGGGGCGCCCCGATAACCGGCCTCATTTATATAGTCCGCC
AAGCGCACTCACCAACATTCCACGAAGTGAGCTTGGGTCGTTGCGTTGTACAGCAA
TAACGAAGCTGTGCAATAGCAAGTTAATTTATTTATTTATAATAGAACTATTTAATT
AAAAGTAAGTTATTTTCATTGTGTCTTCAAATATATTAAGTGATTGTGATAACGGTT
AACGGTTGTTAGAGGATTGGT**ACTAGTATGGTGCGCTCCTCCAAG**AACGTCATCA
AGGAGTTCATGCGCTTCAAGGTGCGCATGGAGGGCACCGTGAACGGCCACGAGTT
CGAGATCGAGGGCGAGGGCGAGGGCCGCCCCTACGAGGGCCACAACACCGTGAAG
CTGAAGGTGACCAAGGGCGGCCCCCTGCCCTTCGCCTGGGACATCCTGTCCCCCCA
GTTCCAGTACGGCTCCAAGGTGTACGTGAAGCACCCCGCCGACATCCCCGACTACA

AGAAGCTGTCCTTCCCCGAGGGCTTCAAGTGGGAGCGCGTGATGAACTTCGAGGAC
GGCGGCGTGGTGACCGTGACCCAGGACTCCTCCCTGCAGGACGGCTGCTTCATCTA
CAAGGTGAAGTTCATCGGCGTGAACTTCCCCTCCGACGGCCCCGTAATGCAGAAGA
AGACCATGGGCTGGGAGGCCTCCACCGAGCGCCTGTACCCCCGCGACGGCGTGCTG
AAGGGCGAGATCCACAAGGCCCTGAAGCTGAAGGACGGCGGCCACTACCTGGTGG
AGTTCAAGTCCATCTACATGGCCAAGAAGCCCGTGCAGCTGCCCGGCTACTACTAC
GTGGACTCCAAGCTGGACATCACCTCCCACAACGAGGACTACACCATCGTGGAGCA
GTACGAGCGCACCGAGGGCCGCCACCACCTGTTCCTGTAGGCATGCATGGTGAGCA
AGGGCGAGGAGCTGTTCACCGGGGTGGTGCCCATCCTGGTCGAGCTGGACGGCGA
CGTAAACGGCCACAAGTTCAGCGTGTCCGGCGAGGGCGAGGGCGATGCCACCTAC
GGCAAGCTGACCCTGAAGTTCATCTGCACCACCGGCAAGCTGCCCGTGCCCTGGCC
CACCCTCGTGACCACCCTGACCTACGGCGTGCAGTGCTTCAGCCGCTACCCCGACC
ACATGAAGCAGCACGACTTCTTCAAGTCCGCCATGCCCGAAGGCTACGTCCAGGAG
CGCACCATCTTCTTCAAGGACGACGGCAACTACAAGACCCGCGCCGAGGTGAAGTT
CGAGGGCGACACCCTGGTGAACCGCATCGAGCTGAAGGGCATCGACTTCAAGGAG
GACGGCAACATCCTGGGGCACAAGCTGGAGTACAACTACAACAGCCACAACGTCT
ATATCATGGCCGACAAGCAGAAGAACGGCATCAAGGTGAACTTCAAGATCCGCCA
CAACATCGAGGACGGCAGCGTGCAGCTCGCCGACCACTACCAGCAGAACACCCCC
ATCGGCGACGGCCCCGTGCTGCTGCCCGACAACCACTACCTGAGCACCCAGTCCGC
CCTGAGCAAAGACCCCAACGAGAAGCGCGATCACATGGTCCTGCTGGAGTTCGTG
ACCGCCGCCGGGATCACTCTCGGCATGGACGAGCTGTACAAGTAA**AAGCTT**GCCC
AATACGCAAACCGCCTCTCCCCGCGCGTTGGCCGATTCATTAATGCAGCTGGCACG
ACAGGTTTCCCGACTGGAAAGCGGGCAGTGAGCGCAACGCAATTAATGTGAGTTA
GCTCACTCATTAGGCACCCCAGGCTTTACACTTTATGCTTCCGGCTCGTATGTTGTG
TGGAATTGTGAGCGGATAACAATTTCACACAGGAAACAGCTATGACCATGATTACG
CCAAGCTGCCCTTAGGGCAGCTTCAATTCGCCCTATAGTGAGTCGTATTACAATTCA
CTGGCCGTCGTTTTACAACGTCGTGACTGGGAAAACCCTGGCGTTACCCAACTTAA
TCGCCTTGCAGCACATCCCCCTTTCGCCAGCTGGCGTAATAGCGAAGAGGCCCGCA
CCGATCGCCCTTCCCAACAGTTGCGCAGCCTGAATGGCGAATGGACGCGCCCTGTA
GCGGCGCATTAAGCGCGGCGGGTGTGGTGGTTACGCGCAGCGTGACCGCTACACTT
GCCAGCGCCCTAGCGCCCGCTCCTTTCGCTTTCTTCCCTTCCTTTCTCGCCACGTTCG
CCGGCTTTCCCCGTCAAGCTCTAAATCGGGGGCTCCCTTTAGGGTTCCGATTTAGTG
CTTTACGGCACCTCGACCCCAAAAAACTTGATTAGGGTGATGGTTCACGTAGTGGG
CCATCGCCCTGATAGACGGTTTTTCGCCCTTTGACGTTGGAGTCCACGTTCTTTAAT
AGTGGACTCTTGTTCCAAACTGGAACAACACTCAACCCTATCTCGGTCTATTCTTTT
GATTTATAAGGGATTTTGCCGATTTCGGCCTATTGGTTAAAAAATGAGCTGATTTAA
CAAAAATTTAACGCGAATTTTAACAAAATTCAGGGCGCAAGGGCTGCTAAAGGAA
GCGGAACACGTAGAAAGCCAGTCCGCAGAAACGGTGCTGACCCCGGATGAATGTC
AGCTACTGGGCTATCTGGACAAGGGAAAACGCAAGCGCAAAGAGAAAGCAGGTAG
CTTGCAGTGGGCTTACATGGCGATAGCTAGACTGGGCGGTTTTATGGACAGCAAGC
GAACCGGAATTGCCAGCTGGGGCGCCCTCTGGTAAGGTTGGGAAGCCCTGCAAAG
TAAACTGGATGGCTTTCTTGCCGCCAAGGATCTGATGGCGCAGGGGATCAAGATCT
GATCAAGAGACAGGATGAGGATCGTTTCGCATGATTGAACAAGATGGATTGCACG
CAGGTTCTCCGGCCGCTTGGGTGGAGAGGCTATTCGGCTATGACTGGGCACAACAG
ACAATCGGCTGCTCTGATGCCGCCGTGTTCCGGCTGTCAGCGCAGGGGCGCCCGGT
TCTTTTTGTCAAGACCGACCTGTCCGGTGCCCTGAATGAACTGCAGGACGAGGCAG
CGCGGCTATCGTGGCTGGCCACGACGGGCGTTCCTTGCGCAGCTGTGCTCGACGTT
GTCACTGAAGCGGGAAGGGACTGGCTGCTATTGGGCGAAGTGCCGGGGCAGGATC
TCCTGTCATCCCACCTTGCTCCTGCCGAGAAAGTATCCATCATGGCTGATGCAATGC
GGCGGCTGCATACGCTTGATCCGGCTACCTGCCCATTCGACCACCAAGCGAAACAT
CGCATCGAGCGAGCACGTACTCGGATGGAAGCCGGTCTTGTCGATCAGGATGATCT
GGACGAAGAGCATCAGGGGCTCGCGCCAGCCGAACTGTTCGCCAGGCTCAAGGCG
CGCATGCCCGACGGCGAGGATCTCGTCGTGACCCACGGCGATGCCTGCTTGCCGAA
TATCATGGTGGAAAATGGCCGCTTTTCTGGATTCATCGACTGTGGCCGGCTGGGTG
TGGCGGACCGCTATCAGGACATAGCGTTGGCTACCCGTGATATTGCTGAAGAGCTT
GGCGGCGAATGGGCTGACCGCTTCCTCGTGCTTTACGGTATCGCCGCTCCCGATTC
GCAGCGCATCGCCTTCTATCGCCTTCTTGACGAGTTCTTCTGAATTGAAAAAGGAA

GAGTATGAGTATTCAACATTTCCGTGTCGCCCTTATTCCCTTTTTTGCGGCATTTTGC
CTTCCTGTTTTTGCTCACCCAGAAACGCTGGTGAAAGTAAAAGATGCTGAAGATCA
GTTGGGTGCACGAGTGGGTTACATCGAACTGGATCTCAACAGCGGTAAGATCCTTG
AGAGTTTTCGCCCCGAAGAACGTTTTCCAATGATGAGCACTTTTAAAGTTCTGCTAT
GTGGCGCGGTATTATCCCGTATTGACGCCGGGCAAGAGCAACTCGGTCGCCGCATA
CACTATTCTCAGAATGACTTGGTTGAGTACTCACCAGTCACAGAAAAGCATCTTAC
GGATGGCATGACAGTAAGAGAATTATGCAGTGCTGCCATAACCATGAGTGATAAC
ACTGCGGCCAACTTACTTCTGACAACGATCGGAGGACCGAAGGAGCTAACCGCTTT
TTTGCACAACATGGGGGATCATGTAACTCGCCTTGATCGTTGGGAACCGGAGCTGA
ATGAAGCCATACCAAACGACGAGCGTGACACCACGATGCCTGTAGCAATGGCAAC
AACGTTGCGCAAACTATTAACTGGCGAACTACTTACTCTAGCTTCCCGGCAACAAT
TAATAGACTGGATGGAGGCGGATAAAGTTGCAGGACCACTTCTGCGCTCGGCCCTT
CCGGCTGGCTGGTTTATTGCTGATAAATCTGGAGCCGGTGAGCGTGGGTCTCGCGG
TATCATTGCAGCACTGGGGCCAGATGGTAAGCCCTCCCGTATCGTAGTTATCTACA
CGACGGGGAGTCAGGCAACTATGGATGAACGAAATAGACAGATCGCTGAGATAGG
TGCCTCACTGATTAAGCATTGGTAACTGTCAGACCAAGTTTACTCATATATACTTTA
GATTGATTTAAAACTTCATTTTTAATTTAAAAGGATCTAGGTGAAGATCCTTTTTGA
TAATCTCATGACCAAAATCCCTTAACGTGAGTTTTCGTTCCACTGAGCGTCAGACCC
CGTAGAAAAGATCAAAGGATCTTCTTGAGATCCTTTTTTTCTGCGCGTAATCTGCTG
CTTGCAAACAAAAAAACCACCGCTACCAGCGGTGGTTTGTTTGCCGGATCAAGAGC
TACCAACTCTTTTTCCGAAGGTAACTGGCTTCAGCAGAGCGCAGATACCAAATACT
GTTCTTCTAGTGTAGCCGTAGTTAGGCCACCACTTCAAGAACTCTGTAGCACCGCCT
ACATACCTCGCTCTGCTAATCCTGTTACCAGTGGCTGCTGCCAGTGGCGATAAGTC
GTGTCTTACCGGGTTGGACTCAAGACGATAGTTACCGGATAAGGCGCAGCGGTCGG
GCTGAACGGGGGGTTCGTGCACACAGCCCAGCTTGGAGCGAACGACCTACACCGA
ACTGAGATACCTACAGCGTGAGCTATGAGAAAGCGCCACGCTTCCCGAAGGGAGA
AAGGCGGACAGGTATCCGGTAAGCGGCAGGGTCGGAACAGGAGAGCGCACGAGG
GAGCTTCCAGGGGGAAACGCCTGGTATCTTTATAGTCCTGTCGGGTTTCGCCACCTC
TGACTTGAGCGTCGATTTTTGTGATGCTCGTCAGGGGGGCGGAGCCTATGGAAAAA
CGCCAGCAACGCGGCCTTTTTACGGTTCCTGGCCTTTTGCTGGCCTTTTGCTCACAT
GTTCTTTCCTGCGTTATCCCCTGATTCTGTGGATAACCGTATTACCGCCTTTGAGTG
AGCTGATACCGCTCGCCGCAGCCGAACGACCGAGCGCAGCGAGTCAGTGAGCGAG
GAAGCGGAAG

Note 2 | pMD19-hA4-pylS sequence

CCGCTCGAGCAGCGTCGTGAAAAGAGGCAATGACAAATACAAAACGACGTATGAG
CAGACCCGTCGCCAAGACGGGTCTACCTCTAAGATGATGTCATTTGTTTTTTAAAAC
TAACTCGCTTTACGAGTAGAATTCTACGTGTAAAACATAATCAAGAGATGATGTCA
TTTGTTTTTCAAAACCAAACTCGCTTTACGAGTAGAATTCTACGTGTAAAACACAAT
CAAAAGATGATGTCATTCGTTTTTCAAAACCGAATTTAAGAAATGATGTCATTTGTT
TTTCAAAACCAAACTCGCTTTACGAGCAGAATTCTACGTGTAAAACACAATCAAGA
GATGATGTCATTTGTTTTTCAAAACTGAATGATGTCATTTGTTTTTCAAAACTAAAC
TTGCTTTGCGAGTAGAATTCTACGTGTAAAACACAGTCAAGAGATGATGTCATTTG
TTTTTCAAAACTGAACCGGCTTTACGAGTAGAATTCTACTTGTAAAACATAATCAA
GAGATGATGTCATTTGTTTTTCAAAACTGAACTGGCTTTACGAGTAGAATTCTACGT
GTAAAACATAATCAAGAGATGATGTCATCATTAAACTGATGTCATTTTATACACGA
TTGTTAACATGTTTAATAATGACTAATTTGTTTTTCCAAATTAAACTCGCTTTACGA
GTAGAATTCTACTTGTAACGCACGATTAAGTATGAATCATAAGCTGATGTCATTTGT
TTTCGACATAAAATGTTTATACAATGGAATCTTCTTGTAAATTATCCAAATAATATA
ATTTATCCGATTCTACGTTACATTTAAATTCGTTGTTATCGTACAATTCTTCAGGAC
ACGCCATGTATTGGTCATTTTTAGCGTGCAACCAACGATTGTATTTGACGCCGTCGT
TGGATTGCGTGTTCAGGTTGGCGTACACGTGACTGGGCACGGCTTCTTTTTCCATGG
GACGTCGACTCATCTTGTCACACCTACATCTTACTAATTTCGTAAGTAGATTTTTTTT
TACACGTATAATGTATGTATTCTTTCCTTAATTAACTTATTTTGAAACGAAATAAAT
AGGCTATTAATATTTGGAACTAGGTTGCGGTCAATGTCAATGTCTGTCTCAACTTTA
ATTCAGAATGCCTTGTGTTCCGTAGATGCTATAAATCAATCAAGATGCATCTTGGAT

TGTTGCCAACTCGCAGCTACAAAATTTGTTTCCAAGCCTAAGCATAGTGCTGTACCC
GTTCCCGTGTATTCAAATCCCGTATAATAGTATAATATACTCCGTAAATGTAGTGTC
ACTGCTTGCTGAAATGATATTGCAAGTTCCGTTGGGAATCTTGCCGTTATCAAGCA
ATGCGATATTAGCGGTATGGCGGGAGGGGGACGCGCAGACTCCCTCTGCTGTATTA
CCATATATGGACACAAAACTTCGTGTATTGTACCCTAGCGCGCGATTGGAGGAGAG
TCTGCGGCGGCGGGGCAGGGGCGCCCCGATAACCGGCCTCATTTATATAGTCCGCC
AAGCGCACTCACCAACATTCCACGAAGTGAGCTTGGGTCGTTGCGTTGTACAGCAA
TAACGAAGCTGTGCAATAGCAAGTTAATTTATTTATTTATAATAGAACTATTTAATT
AAAAGTAAGTTATTTTCATTGTGTCTTCAAATATATTAAGTGATTGTGATAACGGTT
AACGGTTGTTAGAGGATTGGT**ACTAGT**CTCGGATCCATGATTACAATGGATTACAA
GGACGACGACGATAAGGATAAAAAGCCACTCAACACGCTGATTTCTGCGACGGGT
CTCTGGATGTCAAGGACAGGAACTATACACAAAATCAAGCACCATGAGGTTTCACG
CTCTAAGATATACATCGAAATGGCCTGCGGCGACCATCTCGTGGTTAACAATTCCA
GATCATCTCGCACCGCACGTGCGTTAAGGCACCATAAATATAGAAAGACGTGCAA
ACGTTGTAGGGTCTCGGACGAGGATTTGAACAAGTTCCTCACCAAAGCTAATGAAG
ATCAAACGTCTGTCAAAGTAAAGGTCGTAAGCGCTCCGACCCGTACGAAAAAGGC
CATGCCCAAGTCTGTAGCTAGGGCCCCTAAACCACTGGAGAACACAGAAGCTGCCC
AAGCACAGCCGAGCGGCTCCAAATTCTCCCCGGCGATCCCTGTGTCAACACAGGAA
TCGGTGAGTGTTCCCGCTTCAGTTTCTACTAGCATTAGCTCCATAAGTACAGGCGCA
ACTGCGTCAGCTCTGGTCAAGGGTAACACCAATCCTATTACGTCCATGTCGGCTCCT
GTACAAGCCTCCGCACCAGCGTTAACAAAATCGCAGACTGACCGCCTTGAGGTCCT
GTTGAACCCTAAGGATGAAATCTCACTGAACTCAGGTAAACCATTCCGTGAATTGG
AGTCTGAACTCTTAAGCAGACGCAAAAAGGACTTGCAACAAATCTACGCTGAAGA
GCGTGAGAACTATTTAGGTAAACTGGAAAGAGAAATCACCCGCTTCTTTGTGGATA
GGGGTTTCCTGGAAATCAAATCTCCTATTCTGATACCATTGGAGTACATAGAAAGA
ATGGGAATCGACAACGATACGGAGCTCAGCAAGCAAATTTTCAGAGTTGACAAGA
ACTTCTGCTTACGCCCGATGCTTGCTCCCAACCTGTACAATTATCTCCGTAAGTTAG
ACAGGGCCCTGCCTGACCCTATCAAAATCTTCGAAATAGGACCTTGTTACAGGAAG
GAGTCAGATGGCAAAGAACACTTGGAAGAGTTCACCATGCTCAATTTTTGCCAGAT
GGGTAGTGGATGTACAAGAGAGAACTTAGAATCAATCATAACTGACTTCCTTAACC
ACCTGGGCATCGACTTTAAGATTGTCGGTGACTCATGTATGGTATATGGAGACACT
CTCGATGTCATGCATGGAGATTTGGAGCTGTCATCAGCAGTGGTTGGTCCTATCCC
ACTTGACAGAGAATGGGGAATTGATAAACCATGGATAGGCGCCGGTTTCGGACTG
GAACGCCTTCTGAAAGTGAAGCATGACTTTAAAAACATCAAGAGGGCAGCAAGGT
CGGAGTCCTATTACAATGGCATCTCAACGAATCTGTAGTG**AAGCTT**GCCCAATACG
CAAACCGCCTCTCCCCGCGCGTTGGCCGATTCATTAATGCAGCTGGCACGACAGGT
TTCCCGACTGGAAAGCGGGCAGTGAGCGCAACGCAATTAATGTGAGTTAGCTCACT
CATTAGGCACCCCAGGCTTTACACTTTATGCTTCCGGCTCGTATGTTGTGTGGAATT
GTGAGCGGATAACAATTTCACACAGGAAACAGCTATGACCATGATTACGCCAAGCT
GCCCTTAGGGCAGCTTCAATTCGCCCTATAGTGAGTCGTATTACAATTCACTGGCCG
TCGTTTTACAACGTCGTGACTGGGAAAACCCTGGCGTTACCCAACTTAATCGCCTTG
CAGCACATCCCCCTTTCGCCAGCTGGCGTAATAGCGAAGAGGCCCGCACCGATCGC
CCTTCCCAACAGTTGCGCAGCCTGAATGGCGAATGGACGCGCCCTGTAGCGGCGCA
TTAAGCGCGGCGGGTGTGGTGGTTACGCGCAGCGTGACCGCTACACTTGCCAGCGC
CCTAGCGCCCGCTCCTTTCGCTTTCTTCCCTTCCTTTCTCGCCACGTTCGCCGGCTTT
CCCCGTCAAGCTCTAAATCGGGGGCTCCCTTTAGGGTTCCGATTTAGTGCTTTACGG
CACCTCGACCCCAAAAAACTTGATTAGGGTGATGGTTCACGTAGTGGGCCATCGCC
CTGATAGACGGTTTTTCGCCCTTTGACGTTGGAGTCCACGTTCTTTAATAGTGGACT
CTTGTTCCAAACTGGAACAACACTCAACCCTATCTCGGTCTATTCTTTTGATTTATA
AGGGATTTTGCCGATTTCGGCCTATTGGTTAAAAAATGAGCTGATTTAACAAAAAT
TTAACGCGAATTTTAACAAAATTCAGGGCGCAAGGGCTGCTAAAGGAAGCGGAAC
ACGTAGAAAGCCAGTCCGCAGAAACGGTGCTGACCCCGGATGAATGTCAGCTACT
GGGCTATCTGGACAAGGGAAAACGCAAGCGCAAAGAGAAAGCAGGTAGCTTGCAG
TGGGCTTACATGGCGATAGCTAGACTGGGCGGTTTTATGGACAGCAAGCGAACCGG
AATTGCCAGCTGGGGCGCCCTCTGGTAAGGTTGGGAAGCCCTGCAAAGTAAACTGG
ATGGCTTTCTTGCCGCCAAGGATCTGATGGCGCAGGGGATCAAGATCTGATCAAGA
GACAGGATGAGGATCGTTTCGCATGATTGAACAAGATGGATTGCACGCAGGTTCTC

CGGCCGCTTGGGTGGAGAGGCTATTCGGCTATGACTGGGCACAACAGACAATCGG
CTGCTCTGATGCCGCCGTGTTCCGGCTGTCAGCGCAGGGGCGCCCGGTTCTTTTTGT
CAAGACCGACCTGTCCGGTGCCCTGAATGAACTGCAGGACGAGGCAGCGCGGCTA
TCGTGGCTGGCCACGACGGGCGTTCCTTGCGCAGCTGTGCTCGACGTTGTCACTGA
AGCGGGAAGGGACTGGCTGCTATTGGGCGAAGTGCCGGGGCAGGATCTCCTGTCA
TCCCACCTTGCTCCTGCCGAGAAAGTATCCATCATGGCTGATGCAATGCGGCGGCT
GCATACGCTTGATCCGGCTACCTGCCCATTCGACCACCAAGCGAAACATCGCATCG
AGCGAGCACGTACTCGGATGGAAGCCGGTCTTGTCGATCAGGATGATCTGGACGA
AGAGCATCAGGGGCTCGCGCCAGCCGAACTGTTCGCCAGGCTCAAGGCGCGCATG
CCCGACGGCGAGGATCTCGTCGTGACCCACGGCGATGCCTGCTTGCCGAATATCAT
GGTGGAAAATGGCCGCTTTTCTGGATTCATCGACTGTGGCCGGCTGGGTGTGGCGG
ACCGCTATCAGGACATAGCGTTGGCTACCCGTGATATTGCTGAAGAGCTTGGCGGC
GAATGGGCTGACCGCTTCCTCGTGCTTTACGGTATCGCCGCTCCCGATTCGCAGCGC
ATCGCCTTCTATCGCCTTCTTGACGAGTTCTTCTGAATTGAAAAAGGAAGAGTATG
AGTATTCAACATTTCCGTGTCGCCCTTATTCCCTTTTTTGCGGCATTTTGCCTTCCTG
TTTTTGCTCACCCAGAAACGCTGGTGAAAGTAAAAGATGCTGAAGATCAGTTGGGT
GCACGAGTGGGTTACATCGAACTGGATCTCAACAGCGGTAAGATCCTTGAGAGTTT
TCGCCCCGAAGAACGTTTTCCAATGATGAGCACTTTTAAAGTTCTGCTATGTGGCGC
GGTATTATCCCGTATTGACGCCGGGCAAGAGCAACTCGGTCGCCGCATACACTATT
CTCAGAATGACTTGGTTGAGTACTCACCAGTCACAGAAAAGCATCTTACGGATGGC
ATGACAGTAAGAGAATTATGCAGTGCTGCCATAACCATGAGTGATAACACTGCGGC
CAACTTACTTCTGACAACGATCGGAGGACCGAAGGAGCTAACCGCTTTTTTGCACA
ACATGGGGGATCATGTAACTCGCCTTGATCGTTGGGAACCGGAGCTGAATGAAGCC
ATACCAAACGACGAGCGTGACACCACGATGCCTGTAGCAATGGCAACAACGTTGC
GCAAACTATTAACTGGCGAACTACTTACTCTAGCTTCCCGGCAACAATTAATAGAC
TGGATGGAGGCGGATAAAGTTGCAGGACCACTTCTGCGCTCGGCCCTTCCGGCTGG
CTGGTTTATTGCTGATAAATCTGGAGCCGGTGAGCGTGGGTCTCGCGGTATCATTG
CAGCACTGGGGCCAGATGGTAAGCCCTCCCGTATCGTAGTTATCTACACGACGGGG
AGTCAGGCAACTATGGATGAACGAAATAGACAGATCGCTGAGATAGGTGCCTCAC
TGATTAAGCATTGGTAACTGTCAGACCAAGTTTACTCATATATACTTTAGATTGATT
TAAAACTTCATTTTTAATTTAAAAGGATCTAGGTGAAGATCCTTTTTGATAATCTCA
TGACCAAAATCCCTTAACGTGAGTTTTCGTTCCACTGAGCGTCAGACCCCGTAGAA
AAGATCAAAGGATCTTCTTGAGATCCTTTTTTTCTGCGCGTAATCTGCTGCTTGCAA
ACAAAAAAACCACCGCTACCAGCGGTGGTTTGTTTGCCGGATCAAGAGCTACCAAC
TCTTTTTCCGAAGGTAACTGGCTTCAGCAGAGCGCAGATACCAAATACTGTTCTTCT
AGTGTAGCCGTAGTTAGGCCACCACTTCAAGAACTCTGTAGCACCGCCTACATACC
TCGCTCTGCTAATCCTGTTACCAGTGGCTGCTGCCAGTGGCGATAAGTCGTGTCTTA
CCGGGTTGGACTCAAGACGATAGTTACCGGATAAGGCGCAGCGGTCGGGCTGAAC
GGGGGGTTCGTGCACACAGCCCAGCTTGGAGCGAACGACCTACACCGAACTGAGA
TACCTACAGCGTGAGCTATGAGAAAGCGCCACGCTTCCCGAAGGGAGAAAGGCGG
ACAGGTATCCGGTAAGCGGCAGGGTCGGAACAGGAGAGCGCACGAGGGAGCTTCC
AGGGGGAAACGCCTGGTATCTTTATAGTCCTGTCGGGTTTCGCCACCTCTGACTTGA
GCGTCGATTTTTGTGATGCTCGTCAGGGGGGCGGAGCCTATGGAAAAACGCCAGCA
ACGCGGCCTTTTTACGGTTCCTGGCCTTTTGCTGGCCTTTTGCTCACATGTTCTTTCC
TGCGTTATCCCCTGATTCTGTGGATAACCGTATTACCGCCTTTGAGTGAGCTGATAC
CGCTCGCCGCAGCCGAACGACCGAGCGCAGCGAGTCAGTGAGCGAGGAAGCGGAA
G

Note 3 | pUC57-U6-pylT sequence

TCGCGCGTTTCGGTGATGACGGTGAAAACCTCTGACACATGCAGCTCCCGGAGACG
GTCACAGCTTGTCTGTAAGCGGATGCCGGGAGCAGACAAGCCCGTCAGGGCGCGT
CAGCGGGTGTTGGCGGGTGTCGGGGCTGGCTTAACTATGCGGCATCAGAGCAGATT
GTACTGAGAGTGCACCATATGCGGTGTGAAATACCGCACAGATGCGTAAGGAGAA
AATACCGCATCAGGCGCCATTCGCCATTCAGGCTGCGCAACTGTTGGGAAGGGCGA
TCGGTGCGGGCCTCTTCGCTATTACGCCAGCTGGCGAAAGGGGGATGTGCTGCAAG
GCGATTAAGTTGGGTAACGCCAGGGTTTTCCCAGTCACGACGTTGTAAAACGACGG

CCAGTGAATTCGAGCTCGGTACCTCGCGAATGCATCTAGAT**GGCGCGCC**AACTAG
CCATGGAGCTGTCCAAGGAATGCGTAGCAGCTTTCTCCAGCAATACATTTCAAACG
CCTCAATCTTTTTGCGTTCCTTTTTCCTGAGACACCAAGTCTCCTAAAGTCATGATG
ATTGACCTAAAAGAATCAATACAGTTTAATAAATTTATAAGTATTAGGTTATGTAG
TACACATTGTTGTAAATCACTGAATTGTTTTAGATGATTTTAACAATTAGTACTTAT
TAATATTAAATAAGTACATACCTTGAGAATTTAAAAATCGTCAACTATAAGCCATA
CGAATTTAAGCTTGGTACTTGGCTTATAGATAAGGACAGAATAAGAATTGTTAACG
TGTAAGACAAGGTCAGATAGTCATAGTGATTTTGTCAAAGTAATAACAGATGGCGC
TGTACAAACCATAACTGTTTTCATTTGTTTTTATGGATTTTATTACAAATTCTAAAG
GTTTTATTGTTATTATTTAATTTCGTTTTAATTATATTATATATCTTTAATAGAATAT
GTTAAGAGTTTTTGCTCTTTTTGAATAATCTTTGTAAAGTCGAGTGTTGTTGTAAAT
CACGCTTTCAATAGTTTAGTTTTTTTAGGTATATATACAAAATATCGTGCTCTACAA
GT**GGAAACCTGATCATGTAGATCGAATGGACTCTAAATCCGTTCAGCCGGGTT**
AGATTCCCGGGGTTTCCGTTTTTTTGTTTTAGAGCTAGAAATAGCAAGTTAAAAT
AAGGCTAGTCCGTTATCAACTTGAAAAAGTGGCACCGAGTCGGTGCTTTTTTTCTA
GAACAATTTTATAACATACATCGGATTTTTTAATTAGTTTAAAAATATATTTGATTC
GTTATCAAATGTTAACATAAATATTAATACTAGATAAACAGTTTATGTATAAAAAA
TTGTTTATTTTTTTAAATAAAAAAACAAATATTATCCTATTTTTGGTCAAGCTTTTGC
TTTTGGCTAAATCGATAAAGATCTTTCATT**GGCGCGCC**ATCGGATCCCGGGCCCGT
CGACTGCAGAGGCCTGCATGCAAGCTTGGCGTAATCATGGTCATAGCTGTTTCCTG
TGTGAAATTGTTATCCGCTCACAATTCCACACAACATACGAGCCGGAAGCATAAAG
TGTAAAGCCTGGGGTGCCTAATGAGTGAGCTAACTCACATTAATTGCGTTGCGCTC
ACTGCCCGCTTTCCAGTCGGGAAACCTGTCGTGCCAGCTGCATTAATGAATCGGCC
AACGCGCGGGGAGAGGCGGTTTGCGTATTGGGCGCTCTTCCGCTTCCTCGCTCACT
GACTCGCTGCGCTCGGTCGTTCGGCTGCGGCGAGCGGTATCAGCTCACTCAAAGGC
GGTAATACGGTTATCCACAGAATCAGGGGATAACGCAGGAAAGAACATGTGAGCA
AAAGGCCAGCAAAAGGCCAGGAACCGTAAAAAGGCCGCGTTGCTGGCGTTTTTCC
ATAGGCTCCGCCCCCCTGACGAGCATCACAAAAATCGACGCTCAAGTCAGAGGTG
GCGAAACCCGACAGGACTATAAAGATACCAGGCGTTTCCCCCTGGAAGCTCCCTCG
TGCGCTCTCCTGTTCCGACCCTGCCGCTTACCGGATACCTGTCCGCCTTTCTCCCTTC
GGGAAGCGTGGCGCTTTCTCATAGCTCACGCTGTAGGTATCTCAGTTCGGTGTAGG
TCGTTCGCTCCAAGCTGGGCTGTGTGCACGAACCCCCCGTTCAGCCCGACCGCTGC
GCCTTATCCGGTAACTATCGTCTTGAGTCCAACCCGGTAAGACACGACTTATCGCC
ACTGGCAGCAGCCACTGGTAACAGGATTAGCAGAGCGAGGTATGTAGGCGGTGCT
ACAGAGTTCTTGAAGTGGTGGCCTAACTACGGCTACACTAGAAGAACAGTATTTGG
TATCTGCGCTCTGCTGAAGCCAGTTACCTTCGGAAAAAGAGTTGGTAGCTCTTGAT
CCGGCAAACAAACCACCGCTGGTAGCGGTGGTTTTTTTGTTTGCAAGCAGCAGATT
ACGCGCAGAAAAAAAGGATCTCAAGAAGATCCTTTGATCTTTTCTACGGGGTCTGA
CGCTCAGTGGAACGAAAACTCACGTTAAGGGATTTTGGTCATGAGATTATCAAAAA
GGATCTTCACCTAGATCCTTTTAAATTAAAAATGAAGTTTTAAATCAATCTAAAGTA
TATATGAGTAAACTTGGTCTGACAGTTACCAATGCTTAATCAGTGAGGCACCTATC
TCAGCGATCTGTCTATTTCGTTCATCCATAGTTGCCTGACTCCCCGTCGTGTAGATA
ACTACGATACGGGAGGGCTTACCATCTGGCCCCAGTGCTGCAATGATACCGCGAGA
CCCACGCTCACCGGCTCCAGATTTATCAGCAATAAACCAGCCAGCCGGAAGGGCCG
AGCGCAGAAGTGGTCCTGCAACTTTATCCGCCTCCATCCAGTCTATTAATTGTTGCC
GGGAAGCTAGAGTAAGTAGTTCGCCAGTTAATAGTTTGCGCAACGTTGTTGCCATT
GCTACAGGCATCGTGGTGTCACGCTCGTCGTTTGGTATGGCTTCATTCAGCTCCGGT
TCCCAACGATCAAGGCGAGTTACATGATCCCCCATGTTGTGCAAAAAAGCGGTTAG
CTCCTTCGGTCCTCCGATCGTTGTCAGAAGTAAGTTGGCCGCAGTGTTATCACTCAT
GGTTATGGCAGCACTGCATAATTCTCTTACTGTCATGCCATCCGTAAGATGCTTTTC
TGTGACTGGTGAGTACTCAACCAAGTCATTCTGAGAATAGTGTATGCGGCGACCGA
GTTGCTCTTGCCCGGCGTCAATACGGGATAATACCGCGCCACATAGCAGAACTTTA
AAAGTGCTCATCATTGGAAAACGTTCTTCGGGGCGAAAACTCTCAAGGATCTTACC
GCTGTTGAGATCCAGTTCGATGTAACCCACTCGTGCACCCAACTGATCTTCAGCATC
TTTTACTTTCACCAGCGTTTCTGGGTGAGCAAAAACAGGAAGGCAAAATGCCGCAA
AAAAGGGAATAAGGGCGACACGGAAATGTTGAATACTCATACTCTTCCTTTTTCAA
TATTATTGAAGCATTTATCAGGGTTATTGTCTCATGAGCGGATACATATTTGAATGT

ATTTAGAAAAATAAACAAATAGGGGTTCCGCGCACATTTCCCCGAAAAGTGCCACC
TGACGTCTAAGAAACCATTATTATCATGACATTAACCTATAAAAATAGGCGTATCA
CGAGGCCCTTTCGTC
